# ICU infection surveillance can be based on electronic routine data: results of a case study

**DOI:** 10.1186/s12879-023-08082-6

**Published:** 2023-03-01

**Authors:** Tiffany Schaumburg, Norbert Köhler, Yasmine Breitenstein, Susanne Kolbe-Busch, Dirk Hasenclever, Iris F. Chaberny

**Affiliations:** 1 Institute of Hygiene, Hospital Epidemiology and Environmental Health, University of Leipzig Medical Center, Liebigstraße 22, 04103 Leipzig, Germany; 2 Faculty of Medicine, Clinical Trial Centre (ZKS Leipzig), Leipzig University, Leipzig, Germany; 3 Faculty of Medicine, Institute of Medical Informatics, Statistics and Epidemiology (IMISE), Leipzig University, Leipzig, Germany

**Keywords:** Infection prevention and control, Surveillance, Intensive care unit, Nosocomial infections, Automated, Data

## Abstract

**Background:**

The surveillance of hospital-acquired infections in Germany is usually conducted via manual chart review; this, however, proves resource intensive and is prone to a certain degree of subjectivity. Documentation based on electronic routine data may present an alternative to manual methods. We compared the data derived via manual chart review to that which was derived from electronic routine data.

**Methods:**

Data used for the analyses was obtained from five of the University of Leipzig Medical Center’s (ULMC) ICUs. Clinical data was collected according to the Protection against Infection Act (IfSG); documentation thereof was carried out in hospital information systems (HIS) as well as in the ICU-KISS module provided by the National Reference Center for the Surveillance of Nosocomial Infections (NRZ). Algorithmically derived data was generated via an algorithm developed in the EFFECT study; ward-movement data was linked with microbiological test results, generating a data set that allows for evaluation as to whether or not an infection was ICU-acquired.

**Results:**

Approximately 75% of MDRO cases and 85% of cases of sepsis/primary bacteremia were classified as ICU-acquired by both manual chart review and EFFECT. Most discrepancies between the manual and algorithmic approaches were due to differentiating definitions regarding the patients’ time at risk for acquiring MDRO/bacteremia.

**Conclusions:**

The concordance between manual chart review and algorithmically generated data was considerable. This study shows that hospital infection surveillance based on electronically generated routine data may be a worthwhile and sustainable alternative to manual chart review.

**Supplementary Information:**

The online version contains supplementary material available at 10.1186/s12879-023-08082-6.

## Introduction

Hospital-acquired infections are among the most common adverse events in medical care; not only can they lead to significant patient morbidity and mortality, but they may also have a large impact on hospital resources [1–3]. As patients on intensive care units (ICUs) are considered particularly vulnerable for the acquisition of nosocomial infections, developing strategies to track [4] and prevent them has been of particular research interest [5]. One approach in preventing nosocomial infections is the reduction of microorganisms and their reservoirs by means of antiseptic body wash.

Given this background, we conducted a trial assessing the effect of daily antiseptic body wash with octenidine on nosocomial primary bacteremia and the acquisition of nosocomial multidrug-resistant organisms (MDROs) on ICUs [6].

Unlike previous trials [7, 8], the EFFECT endpoints (ICU-acquired primary bacteremia and ICU-acquired MDROs) were not based on manual chart review of sepsis-related and MDRO-related events but rather on routine data obtained from hospital and laboratory information systems (HIS/LIS); the endpoints were derived using an algorithm which linked both ward-movement data and microbiological data for each individual ICU patient [6].

In this case study, we compare the results obtained using the EFFECT algorithm to the results obtained from manual clinical documentation as conducted according to the German Protection against Infection Act (IfSG, §4 Sec. 2 and §23, Sec. 4).

The main objective of this study was to investigate how the results obtained using the EFFECT algorithm compare to those obtained via manual chart review and clinical documentation. We will also discuss whether routine data from HIS/LIS can be used for monitoring nosocomial infections.

## Materials and methods

### Data acquisition

Data used for the analyses was obtained from five of the University of Leipzig Medical Center’s (ULMC) ICUs and covers a period of 12 months (April 2017 to March 2018). While the clinical documentation data set was collected by medical staff according to the Protection against Infection Act (IfSG) and recorded in both the HIS and the ICU-KISS module, the EFFECT data set was extracted from HIS and LIS [6].

As part of the clinical documentation data set, the infection prevention team used data forwarded to them from the microbiological lab in order to assess and internally document multidrug-resistant pathogens in the HIS; this approach is compliant with the IfSG and can be used for internal quality assurance within the individual hospital. The ICU-KISS module, on the other hand, focuses not only on microbiological data but on patients’ symptoms [9]. Nosocomial infections are evaluated based on the definitions published by the CDC that have been further adapted by the National Reference Center for the Surveillance of Nosocomial Infections (NRZ); this approach is used to generate reference data throughout Germany which is suitable for public use throughout the country and the European Union. ICU-KISS data was used to quantify the number of clinically documented ICU-acquired sepsis cases during the aforementioned time period.

In contrast to the clinical documentation approach, we analyzed the same time period based on routine data from HIS and LIS using the EFFECT algorithm. By linking individual ward-movement history (that includes information such as timestamps for admission and discharge) with microbiological test results (that include information on the type of specimen, date of sample collection, detected pathogen and antibiogram), we generated a data set which allowed us to determine whether an infection was ICU-acquired [6].

These two data sets were then compared with regard to the number of identified cases of ICU-acquired primary bacteremia and ICU-acquired MDROs. Both clinical documentation data and data from hospital and laboratory information systems had spreadsheet format and were imported into **R** [10].

### Statistical analyses

Standard methods of descriptive statistics were used indicating frequencies, percentages and measures of central tendency and dispersion (median along with IQR). We used flow diagrams to visualize the consistency between manual and algorithmic documentation. For all statistical analyses, the software environment for statistical computing **R** (version $\ge$ 4.2.0) was used.

## Results

### Sample characteristics

Six thousand, four hundred sixty patients were admitted to the ICU between April 2017 and March 2018. Four hundred ninety-nine patients had more than one ICU treatment episode (maximum: 6 ICU treatment episodes). The total number of ICU treatment episodes was 7350, of which 3327 (45%) had a duration of > 48 h. While the median (IQR) treatment duration of all patients was 1.8 (0.9, 3.9) days, the median (IQR) treatment duration of patients at risk for nosocomial events was 4.3 (3.0, 8.6) days.

The microbiological data contains 41,719 unique laboratory numbers. Overall, the median (IQR) number of laboratory numbers per patient was 3.0 (2.0, 7.0). In 176 patients (2.7%), no laboratory number was documented. As for patients with an ICU treatment duration of > 48 h, the median (IQR) number of laboratory numbers per patient was 6.0 (2.0, 12.0) with a total of 40 patients (1.3%) for whom no laboratory number was documented.

Approximately 35%, 25%, and 15% of the tests can be attributed to nose/throat swabs, rectal/anal swabs and blood samples respectively.

In total, 5822 positive bacterial swabs were detected during the period under observation, of which 3277 were gram-negative (56.3%), 1309 were gram-positive (22.5%), 1157 were common commensals (19.9%) and 79 were “exceptional” (1.4%) [11]. The three most prevalent bacteria were *Escherichia coli* (n = 1289), *Staphylococcus epidermidis* (n = 539) and *Staphylococcus aureus* (n = 497). A total of 192 distinct organisms were found in the data.

### Multidrug-resistant organisms

#### MRSA

**Fig. 1:**
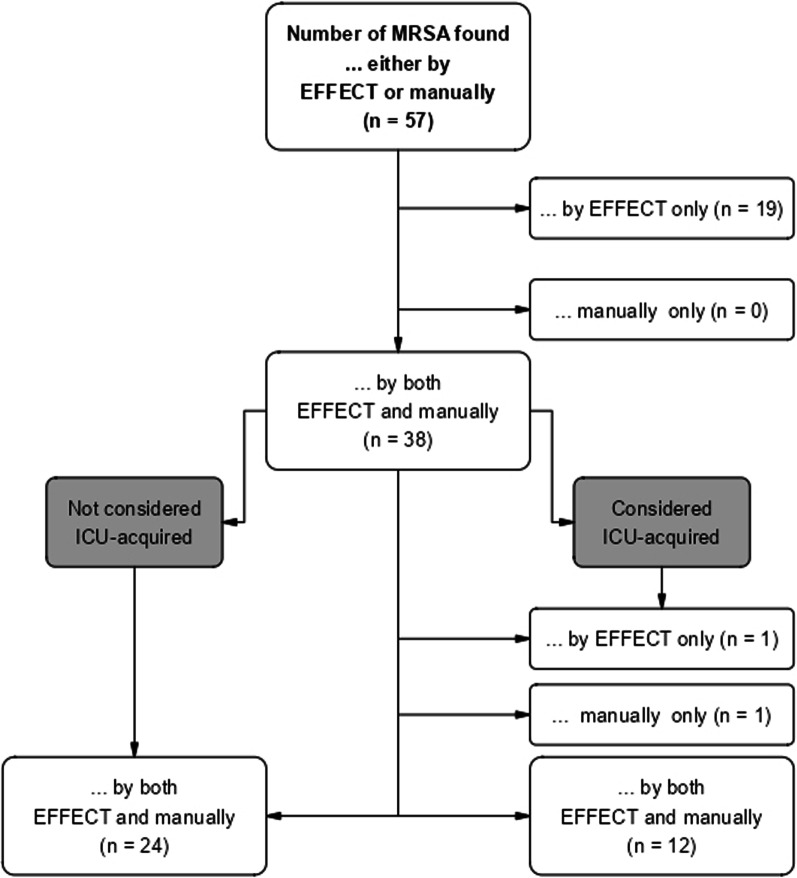
MRSA: EFFECT vs. manual documentation

A total of 57 *Staphylococcus aureus* were considered MRSA by either EFFECT or manual documentation. While 38 were mutually considered MRSA (67%), 19 (33%)/0 were considered MRSA by EFFECT/manual documentation only (see Fig. 1). Of the 19 MRSA detected by EFFECT only, 17 (89%) were detected before ICU admission.

1. *Total number of MRSA identified as possibly ICU-acquired by ****EITHER**** EFFECT or manually on all 5 ICUs between April 2017 and March 2018 (n* = *57).*
2. *How many of these 57 MRSA were identified by ****BOTH**** EFFECT and manually? (n* = *38)*
  - *19 MRSA were found by EFFECT only; this is due to detection prior to ICU admission (n* = *17) and detection between ICU admission and day two after ICU admission (n* = *2)*
3. *How many of these 38 MRSA were considered…*
  - *ICU-acquired? (n* = *14, right-hand side of the diagram)*
    i …*.by EFFECT only (n* = *1, due to human error in manual documentation)*
    ii *…manually only (n* = *1, due to human error in manual documentation)*
    iii *…by both EFFECT and manually (n* = *12)*
  - *Non-ICU-acquired? (n* = *24, left-hand side of the diagram)*

Of the 38 MRSA detected by both EFFECT and manual documentation, 12 (32%)/24 (63%) were mutually considered ICU-acquired/not ICU-acquired and 1 (3%)/1 (3%) were considered ICU-acquired by EFFECT/manual documentation only. In 36 out of 38 total cases (95%), EFFECT and manual documentation were concordant.

In the one case where EFFECT and manual documentation were discordant, the MRSA was detected between days one and two after ICU admission and is therefore not considered ICU-acquired according to EFFECT. In the other case, the MRSA was incorrectly classified as non-ICU-acquired by manual documentation.

#### VRE

**Fig. 2:**
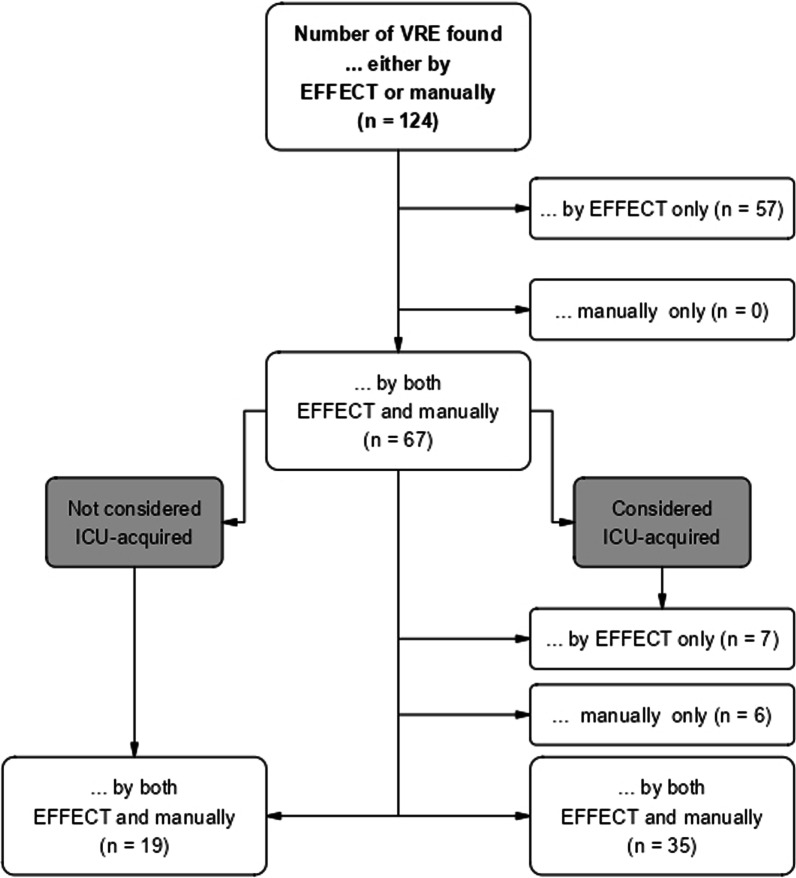
VRE: EFFECT vs. manual documentation

A total of 124 *E. faecium*/*E. faecalis* isolates were considered VRE by either EFFECT or manual documentation. While 67 were mutually considered VRE (54%), 57 (46%)/0 were considered VRE by EFFECT/manual documentation only (see Fig. 2). Of the 57 VRE only detected by EFFECT, 56 (98%) were detected either before ICU admission or up to two days after ICU discharge.

Of the 67 VRE detected by both EFFECT and manual documentation, 35 (52%)/19 (28%) were mutually considered ICU-acquired/not ICU-acquired, and 7 (10%)/6 (9%) were considered ICU-acquired by EFFECT/manual documentation only. In 54 out of 67 total cases (81%), EFFECT and manual documentation were concordant.

In three out of six cases where the VRE were considered ICU-acquired via manual documentation only, it was due to differences in definition of the time at risk for nosocomial events (see Additional file 1: 5.3). In two cases, the VRE was detected before ICU admission and should not have been considered ICU-acquired. And in the remaining case, the VRE was detected one month earlier by EFFECT when compared to manual documentation.

In five out of seven cases when the VRE were considered ICU-acquired by EFFECT only, ULMC received information about prior findings from external hospitals where the patient was treated prior to ICU admission. This information was not part of the microbiological data set and could therefore not be taken into account by EFFECT. In two further cases, the different results can be attributed to significant differences in the day the VRE was detected.

#### MDRGN

**Fig. 3:**
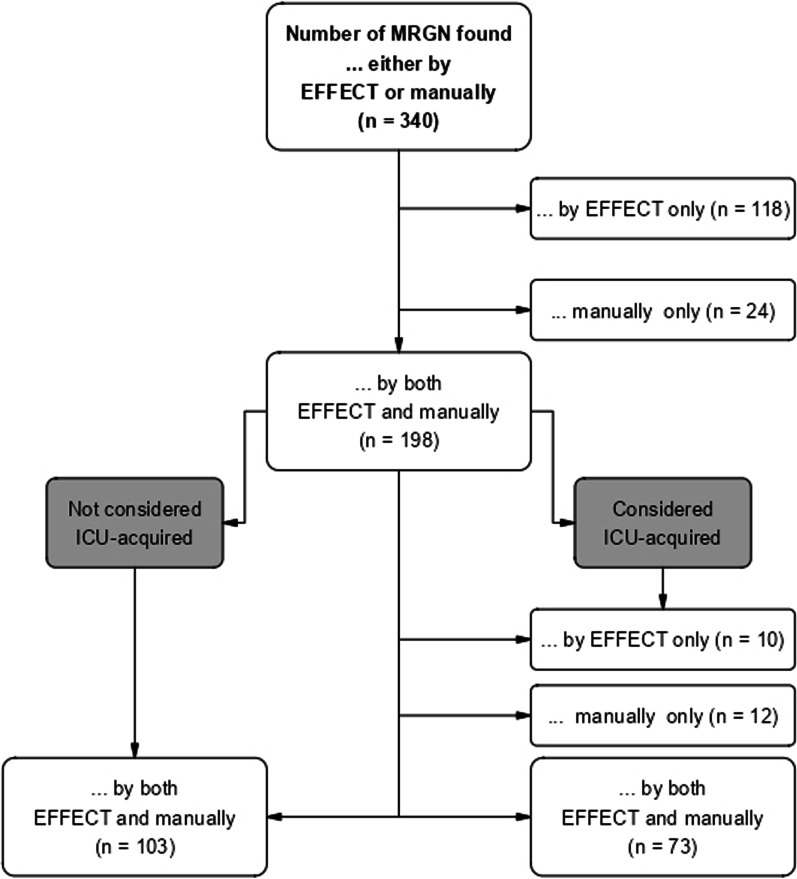
MDRGN: EFFECT vs. manual documentation

A total of 340 gram-negative microorganisms were considered MDRGN by either EFFECT or manual documentation. While 198 were mutually considered MDRGN (58%), 118 (35%)/24 (7%) were considered MDRGN by EFFECT/manual documentation only (see Fig. 3). Of the 118 MDRGN detected by EFFECT only, 94 (80%) were detected either before ICU admission or up to 2 days after ICU discharge. For 16 out of 24 MDRGN detected by manual documentation only, no antibiogram was available; in seven cases, the antibiogram did not indicate multidrug-resistance, and in one case the MDRGN was not documented in the microbiological data.

Of the 198 MDRGN detected by both EFFECT and manual documentation, 73 (37%)/103 (52%) were mutually considered ICU-acquired/not ICU-acquired and 10 (5%)/12 (6%) were considered ICU-acquired by EFFECT/manual documentation only. In a total of 176/198 cases (89%), EFFECT and manual documentation were concordant.

In six out of ten cases where the MDRGN was considered ICU-acquired by EFFECT only, ULMC received information about prior findings from external hospitals where the patient was treated prior to ICU admission. This information was not part of the microbiological data set and could therefore not be taken into account by EFFECT. In the four remaining cases, the MDRGN should have been classified as ICU-acquired via manual documentation.

In two out of twelve cases where the MDRGN was considered ICU-acquired via manual documentation only, EFFECT and manual documentation came to different conclusions due to significant differences in the date the organism was considered multi-resistant. In three further cases, EFFECT did not consider the MDRGN ICU-acquired, because the organisms were detected on day two after ICU admission. In the remaining seven cases, the MDRGN should not have been classified ICU-acquired via manual documentation, as they were detected before the time at risk (up to day one after ICU admission).

### Primary bacteremia/sepsis

During the period under observation, 94 ICU-acquired primary sepses were documented using ICU-KISS. The following analyses show how primary sepses considered ICU-acquired by ICU-KISS were classified by EFFECT (using definitions a and b for common commensals; see Additional file 1: 5.2).

Depending on the definition of bacteremia with common commensal organisms, EFFECT found 202 (definition a) and 117 (definition b) cases of ICU-acquired primary bacteremia.

In total, 94 primary sepses were considered ICU-acquired by ICU-KISS. Of these, 80 (85.1%) were considered primary bacteremia, 8 (8.5%) were considered secondary bacteremia and 6 (6.4%) were considered no bacteremia by EFFECT.

In five out of six bacteremia cases considered primary sepsis by ICU-KISS and no bacteremia by EFFECT, a negative/non-positive blood test was found by EFFECT up to two days after the positive blood culture. In the one remaining case, the microorganism identified as causing primary sepsis by manual documentation via ICU-KISS was not documented in the microbiological data set used by EFFECT.

Eight cases of primary sepsis (according to ICU-KISS) were considered secondary bacteremia by EFFECT. In all cases, the same organism found in a blood culture was also found in other relevant material (therefore making a case for secondary bacteremia as opposed to primary).

#### Pathogenic organisms

A total of 67 ICU-acquired cases of primary bacteremia with pathogenic microorganisms were found by EFFECT, and 49 ICU-acquired primary sepses with pathogenic microorganisms were documented by ICU-KISS. The number of distinct pathogenic microorganisms found by EFFECT (n = 23) was greater than the number documented by ICU-KISS (n = 15). The three most prevalent pathogenic organisms found to be the cause of ICU-acquired primary bacteremia (EFFECT)/sepsis (manual documentation/ICU-KISS) were *Staphylococcus aureus* (n = 14/11), *Enterococcus faecium* (n = 13/10) and *Enterococcus faecalis* (n = 9/8).

#### Common commensals

As specified in Additional file 1: 5.2, EFFECT uses two different definitions for primary bacteremia with common commensal organisms.

While 135 and 52 ICU-acquired primary bacteremia with common commensal organisms were found by EFFECT by applying definitions (a) and (b) respectively, 45 ICU-acquired primary sepses with common commensal organisms were documented in ICU-KISS. The number of distinct common commensal organisms detected was 16 (EFFECT, definition a), 10 (EFFECT, definition b) and 8 (ICU-KISS). Table 1 shows the three most prevalent common commensal organisms found to be the cause of ICU-acquired primary bacteremia/sepsis.

**Table 1:** Top three common commensals: EFFECT vs. manual documentation (ICU-KISS)

Organism	EFFECT		Manual documentation
	Def. a^1^	Def. b^2^	ICU-KISS
*Staphylococcus epidermidis*	58	23	22
*Cutibacterium acnes*	33	13	11
*Staphylococcus capitis*	10	5	5

^1^Single positive blood sample with the respective organism with no further blood samples within the next 2 days

^2^At least one additional confirmatory blood sample with the same respective organism within the next 2 days

## Discussion

This study’s goal was to compare manual clinical documentation to an algorithm developed for the EFFECT trial in an effort to streamline infection surveillance. While manual documentation is time consuming, resource intensive and prone to a certain degree of subjectivity, especially in formats such as ICU-KISS, the use of electronic routine data may help to save costs and could relieve clinical staff from excess clinical documentation [4].

The results of this study indicate considerable concordance between manual documentation and the EFFECT algorithm. Approximately 75% of MDRO cases and 85% of cases of primary bacteremia/sepsis were classified as ICU-acquired by both manual chart review and EFFECT. The rates for MDROs can be calculated as follows: for each flow chart figure (MRSA, VRE, MDRGN), the lower right-hand corner should be consulted (= “considered ICU-acquired”). A quotient is then built with the number identified *by both EFFECT and manually* divided by the sum of *by EFFECT only, manually only* and *by both EFFECT and manually.* An example using the MDRGN flow chart figure is as follows: 73/(73 + 12 + 10) = 0.76 × 100 = 76%. This is done for MRSA, VRE and MDRGN in order to reach a conclusion of approximately 75% across all MDROs. Most discrepancies in the number of identified nosocomial infections could be attributed to different definitions for the time at risk regarding endpoint acquisition; other reasons for data discordance were misclassifications that happened during manual chart review, or information about prior/external test results being available for clinical staff only via document transfer but not for EFFECT’s data sets.

The findings of this study suggest that the surveillance of MDROs and bacteremia is not bound to a conventional manual clinical documentation workflow. Information about nosocomial infections can be derived algorithmically from data provided by HIS and LIS instead of painstakingly via manual chart review. The algorithmic approach is also flexible regarding different endpoint definitions and can be formatted to fit the needs of the observer.

What is more, additional information important for quality assurance can easily be obtained from electronic routine data, such as whether or not screening practices are being adhered to. This ensures a higher level of quality hospital-wide, not only in high-risk environments such as the ICU.

## Conclusions

Innovation and forward thinking are needed when it comes to infection prevention and control; this study shows that routine data can be implemented in order to conduct high-quality yet sustainable surveillance.

By implementing an algorithmic approach to infection surveillance, a health care facility and its infection prevention and control team stand to gain not only a better overview of the happenings on high-risk wards but time and resources, which can be recommitted to other activities that actively foster infection prevention.

## Supplementary Information

**Additional file 1:** Definitions and additional information regarding multidrug-resistant organisms (MDROs), primary bacteremia/sepsis and time at risk.

## Data Availability

The datasets generated and/or analyzed during the current study are not publicly available due to patient privacy regulations but are available from the corresponding author on reasonable request.
