# Supplementary material for: ICU infection surveillance can be based on electronic routine data: results of a case study
Source: BMC Infect Dis. 2023 Mar 1;23:126. doi: 10.1186/s12879-023-08082-6 (PMC9979400; doi:10.1186/s12879-023-08082-6)
Supplement: Supplementary file 1 — Additional file 1: Definitions and additional information regarding multidrug-resistant organisms (MDROs), primary bacteremia/sepsis and time at risk. [file 12879_2023_8082_MOESM1_ESM.docx]

Supplement

5 Definitions

5.1 MDRO

The EFFECT-specific detection of MDROs is based on both the antibiogram and the diagnosis as documented by the laboratory (a text string). Both the antibiogram and MDRO-related text strings are part of the microbiological data. MDRO-related text strings were available for MRSA and VRE but not for MDRGN.

The algorithms for detecting MDRO are based on the definitions outlined by the Commission for Hospital Hygiene and Infectious Disease Prevention at the Robert Koch Institute (12, 13):

An **MRSA** is assumed if a *Staphylococcus aureus* is resistant against oxacillin, and a **VRE** is assumed if an *Enterococcus faecalis* or *Enterococcus faecium* is resistant against vancomycin. In the antibiogram, resistance is indicated by both letters “R” and “I”. In addition, we assume resistance to methicillin (oxacillin)/vancomycin if the tag “MRSA”/“VRE” is found in the data.

In order to identify **MDRGN**, four groups of antibiotics are relevant:

- Ureidopenicillins (lead substance: piperacillin)
- Third and fourth generation cephalosporins (lead substances: cefotaxim, ceftazidim)
- Carbapenems (lead substance: imipenem, meropenem) and
- Fluoroquinolones (lead substance: ciprofloxacin)

***Enterobacterales*** are considered MDRGN as long as they are resistant to (1) piperacillin, ciprofloxacin and either cefotaxim or ceftazidim or (2) if they are resistant to imipenem or meropenem. It is possible that a naturally occurring reduced sensitivity to imipenem can be detected among the following pathogens: Proteus spp., Morganella morganii and Providencia spp. Thus, for these bacteria, solely meropenem is used as the lead substance for the carbapenems group. ***Pseudomonas aeruginosa*** is only considered multidrug resistant if it displays a group resistance to at least three out of the four antibiotic groups. Since Pseudomonas aeruginosa is considered intrinsically resistant to cefotaxim, a resistance to ceftazidim indicates a group resistance to 3^rd^ and 4^th^ generation cephalosporins. ***Acinetobacter baumanii*** is intrinsically resistant to piperacillin, ceftazidim and cefotaxim. We consider this pathogen to be multidrug resistant if it displays resistance to ciprofloxacin, imipenem or meropenem. Bacteria belonging to the *Acinetobacter baumannii complex (ACB)*, that is: Acinetobacter calcoaceticus, Acinetobacter dijkshoorniae, Acinetobacter lactucae, Acinetobacter nosocomialis, Acinetobacter pittii, and Acinetobacter seifertii are treated on the same footing as Acinetobacter baumannii. *Burkholderia* species and *Stenotrophomonas maltophilia* are not classified as MDRGN.

5.2 Primary bacteremia/sepsis

With regard to detecting primary bacteremia, EFFECT differentiates between *pathogenic* organisms and *common commensal* organisms.

If a pathogenic organism is detected in a blood culture but not in any other relevant patient samples, EFFECT categorizes this as primary bacteremia.

A primary bacteremia with a common commensal organism is assumed if either

1. there is a single positive blood sample with the respective organism (with no further blood samples within the next two days), or
2. there is a **positive** blood sample with the respective organism with at least **one additional** confirmatory blood sample with the same respective organism within the next two days (regardless of any additional negative blood tests).

Bacteremia is not assumed if a single positive blood test is followed by additional blood samples within two days that are all negative. In an additional analysis, we restrict the definition of bacteremia to cases with definition b) only.

Regarding pathogenic organisms, ICU-KISS uses the same definition as EFFECT; regarding common commensal organisms, ICU-KISS uses the EFFECT definition **b)**. However, unlike the EFFECT definition, the diagnosis of a primary sepsis requires at least one of three clinical symptoms (fever over 38°C, low blood pressure, chills) (9). EFFECT does not take clinical symptoms into account.

5.3 Time at risk

According to EFFECT (Statistical Analysis Plan), a primary bacteremia counts as ICU-acquired if the organism is found in a blood culture between day three after ICU admission and day two after ICU discharge. This definition deviates from the ICU-KISS definition, which counts all bacteremia detected between day two after ICU admission and ICU discharge as ICU-acquired. Both EFFECT and manual documentation do not count bacteremia as ICU-acquired if the same organism found within the time at risk was detected before the time at risk.
